# Prevalence and correlates of depression and alcohol use disorder among adults attending primary health care services in Nepal: a cross sectional study

**DOI:** 10.1186/s12913-018-3034-9

**Published:** 2018-03-27

**Authors:** Nagendra P. Luitel, Emily C. Baron, Brandon A. Kohrt, Ivan H. Komproe, Mark J. D. Jordans

**Affiliations:** 1 Research Department, Transcultural Psychosocial Organization (TPO) Nepal, Kathmandu, Nepal; 2 Alan J. Flisher Centre for Public Mental Health, University of Cape Town, Cape Town, South Africa; 3 Department of Psychiatry, George Washington University, Washington, DC USA; 4 Faculty of Social and Behavioural Sciences, Utrecht University, Utrecht, the Netherlands; 5 Research and Development Department, HealthNet TPO, Amsterdam, Netherlands; 6 Centre for Global Mental Health, Institute of Psychiatry, Psychology and Neuroscience, King’s College London, London, UK

**Keywords:** Depression, Alcohol use disorder, Primary health care, Nepal

## Abstract

**Background:**

Although depression and alcohol use disorder (AUD) are expected to be common among patients presenting to primary health care setting, there is limited research on prevalence of depression and AUD among people attending primary health care services in low-income countries. The aim of this study was to assess the prevalence of depression and AUD among adults attending primary care facilities in Nepal and explore factors associated with depression and AUD.

**Methods:**

We conducted a population-based cross-sectional health facility survey with 1474 adults attending 10 primary healthcare facilities in Chitwan district, Nepal. The prevalence of depression and AUD was assessed with validated Nepali versions of the Patient Health Questionnaire (PHQ-9) and Alcohol Use Disorder Identification Test (AUDIT).

**Results:**

16.8% of the study sample (females 19.6% and males 11.3%) met the threshold for depression and 7.3% (males 19.8% and females 1.1%) for AUD. The rates of depression was higher among females (RR = 1.48, *P* = 0.009), whereas rates of AUD was lower among females (RR = 0.49, *P* = 0.000). Rates of depression and AUD varied based on education, caste/ethnicity, occupations and family income.

**Conclusions:**

In Nepal, one out of five women attending primary care services have depression and one out of five men have AUD. Primary care settings, therefore, are an important setting for detection and treatment initiation for these conditions. Given that “other” occupation is at increased risk for both conditions, it will be important to assure that treatments are feasible and effective for this high risk group.

## Background

Mental, neurological and substance use (MNS) disorders are one of the leading causes of disability worldwide [1], contributing to 14% of the global burden of disease [2]. The magnitude of depression and alcohol use disorder varies across different regions and age groups; however, depression and AUD have been reported to be the second and third leading cause of years lived with disability [3–5]. The recent World Health Survey conducted with 245,404 adults from 60 countries reported a 3.2% ICD-10 prevalence of depression [6]. The prevalence rates of depression were reported to be higher in low and middle income countries (LMICs) such as 5.5% in Nigeria [7], 17.5% in Ethiopia [8], 10.3% in Malaysia [9], 45.9% in Pakistan [10]; 15.1% in India [11] and 17.4% in Uganda [12]. Similarly, AUDs are amongst the most prevalent mental disorders worldwide [13]. For example, the reported prevalence rates for AUD were 9% in Colombia [14], 18.4% in Brazil [15], 6.7% in Thailand [16] and 8.5% in India [17]. The prevalence of mental disorders can be even higher among people attending primary health care facilities [18, 19]; however, a large variation has been found in the reported rates of depression (4.5% to 47.8%) and AUD (8.2% to 28.7%) among primary care attendees [20–26]. Despite the high burden of depression and AUD, it is reported that more than half (56%) of people with depression [27–30] and 78% persons with alcohol abuse and dependence [29] have not received care.

In Nepal, few studies have been conducted to estimate prevalence of depression and alcohol use disorder. Available data show a large variation in reported rates of depression (ranging from 6% to 81%) [31–33] and alcohol use disorder (ranging from 1.5 to 25%) [34–36], however these studies have been conducted with high risk groups (e.g., torture survivors and refugees) or in communities immediately post-conflict. Recent prevalence studies reported the rates of depression (11.7% and 27.5%) and AUD (5%) in the community sample [37, 38]. In clinics treating diabetes and hypertension, two out of five patients scored above locally validated thresholds for depression [39, 40]. As depression and alcohol use disorders are often associated with physical health problems in Nepal [41], there is a possibility that a significant proportion of the patients attending primary health care facilities may have depression and/or alcohol use disorder in Nepal. However, no systematic studies have been conducted yet to estimate prevalence of depression and alcohol use disorder among people attending primary care in Nepal.

In Nepal there is scarcity of population-wide mental health services. Across the country, there are about 440 in-patient beds allocated for mental illness, which accounts to 1.5 beds per 100,000 people. Specialized mental health services are limited to few hospitals located in the large cities with 0.22 psychiatrists and 0.06 psychologists per 100,000 people. Community mental health has only been initiated by non-governmental organizations; however, these services are limited and constrained by issues such as lack of regular supply of medicines and supervision [42].

Evidence has acquired that mental health treatment can be provided in primary and community health care settings by trained health workers using the task sharing approach [43–45]. However, feasibility of detection and initiation of evidence based treatment in primary care have not been widely studied [46]. This study was conducted as a part of PRIME (PRogramme for Improving Mental health carE) research program consortium which aims to generate evidence on implementation and scale up of mental health treatment in primary and maternal health care contexts in low resource settings [46]. The purpose of this study was to estimate the target population and demographic risk factors of the affected population in order to most effectively design and deliver interventions that will reach the populations most in need [47, 48]. This article presents the prevalence of depression and AUD and risk factors for these conditions among adults attending primary health care facilities in southern Nepal.

## Methods

### Setting

Nepal is one of the poorest countries in South-Asia and has a total population of approximately 26.4 million with 69.1 years life expectancy at birth. The United Nations ranks Nepal 145th out of the world’s 188 countries on the Human Development Index (HDI) [49]. Primary health care services in Nepal are provided at the district level through sub-health posts (SHPs), health posts (HPs) and primary health care centers (PHCCs). Sub-health posts are the first institutional contact point for basic health services in the community. Sub-health posts provide essential health care packages and also monitor the activities of female community health volunteers (FCHVs) as well as community-based activities through PHC outreach clinics. Health posts (HP) are the next tier of the health care system and they offer the same package of essential health care services as SHPs, with the additional services of birthing centers. Health posts also monitor the activities of SHPs. SHPs and HPs are staffed by health assistants (HA), auxiliary health workers (AHW), auxiliary nurse mid-wife (ANM) and maternal and child health workers (MCHW). The third tier of health care system is primary health care centers (PHCCs). The services delivered within PHCCs are general medical care, family planning, maternal and child health, basic laboratory investigations and provision of basic health care services that are available in the SHPs and HPs. PHCCs are staffed by medical officer, HAs, AHWs, ANMs and MCHWs [42]. This study was conducted in Chitwan, a southern district in Nepal. The total population of Chitwan is 579,984 (male 279,087 and female 300,897) with about 132,462 households. There are 41 health facilities (2 hospitals, 3 primary health care centers and 36 health posts/sub-health posts) in the Chitwan district. This study was conducted in 10 primary health care facilities (9 health posts/sub-health posts and one primary health care center) in order to most effectively design and deliver the PRIME interventions. Data were collected between September 2013 and February 2014.

### Study design

This study was a population-based cross-sectional health facility survey conducted before implementation of the district Mental Health Care Plan (MHCP).

### Sample size and sampling

The total sample of the study was 1474 adults who attended the 10 primary care clinics. The sample size was determined to allow detection of change in diagnosis of depression and AUD in the primary care clinics between the baseline and subsequent follow up studies, with 80% statistical power and two-sided alpha of 0.05. The expected increases in the detection rate of depression and AUD were 0 to 1% at the baseline to between 20 to 25% in the end-line. The calculated sample size was allocated to the 10 primary health care clinics based on the clients’ flow in each clinic in the past 3 months preceding the survey. We invited any eligible person attending the health facility for the study. When there was more than one person attending simultaneously, we randomly selected one person. For this purpose, first, the field workers prepared a list of all eligible participants when entering the clinic. Second, a member of the group drew a name randomly from the list by using a piece of paper. Finally, field workers conducted the interview with the selected participant while waiting for services. As the client flow was very low in most of the health facilities i.e. most of the time only one participant visited clinics at any given time, in most of the cases we recruited participants individually and randomization was not needed. The inclusion criteria for participation in the study included age of 18 years or above, fluency in Nepali language, time and availability to complete full survey which was administered orally by research assistants to the respondents, and willingness to provide informed consent. Exclusion criteria were incapability to provide informed consent, currently experiencing an acute medical issue. Of the total invited participants, 58 participants did not agree to participate or dropped out from the interviews.

### Data collection

The interviewers were 12 Nepali speaking research assistants who had completed at least an undergraduate degree. The research assistants received one month training covering the topics of interviewing skills, rapport building, informed consent, ethical consideration, inclusion/exclusion criteria, content of the questionnaire, as well as field testing. Android tablets with a questionnaire application were used for data collection [50]. Interviews were conducted in separate rooms within the health facilities or in the ground within the primary health care facilities. Data collection was done in two parts: before the consultation with PHC workers (screening and treatment history) and post consultation to assess if diagnosis of depression or AUD was provided or not.

### Instruments

We assessed depression, Alcohol Use Disorder (AUD) and treatment history for both depression and AUD. Details of the instruments have been presented elsewhere; in this section we have described the tools which are related to this paper.

#### Patient health questionnaire (PHQ9)

The PHQ9 is a self-report screening tool designated for use in various medical settings. PHQ9 has been widely used and validated in primary care, medical outpatients, and specialist medical services [51]. It has 9-items and the respondents were asked to score nine common symptoms of depression in the past 2 weeks. It has a 4-points rating scale from 0 ‘not at all’ to 3 ‘always’. The PHQ9 has been translated and validated in a primary care population in Chitwan, Nepal: the validated cutoff score of ≥10 (sensitivity =0.94, specificity = 0.80, positive predictive value (PPV) = 0.42, negative predictive value (NPV) = 0.99, positive likelihood ratio = 4.62 and negative likelihood ratio = 0.07) has been recommended for moderate to severe depression symptom [52]. After completing the PHQ9, each participant was also asked an additional question to assess depressive episodes in the past 12 month period.

#### Alcohol use disorder identification test (AUDIT)

The AUDIT was developed by the World Health Organization (WHO) as a screening tool for alcohol use disorder in primary health care [53]. The AUDIT has been validated in Nepal using DSM-IV diagnostic categories (alcohol use and alcohol dependence) as the gold standard to calculate the diagnostic parameters of the AUDIT, and a cutoff score of ≥9 has been recommended for alcohol dependence or alcohol abuse for both males (sensitivity 96.7, specificity 91.7, PPV 90.3 and NPV 97.2) and females (sensitivity 94.3, specificity 91.4, PPV 80.1 and NPV 97.8) [54].

### Data analysis

Descriptive statistics were used to report on the demographic characteristics of the sample. We presented age, sex, education, caste/ethnicity, occupation, marital status, religion and number of family members in the household. Second, we presented percentages of the participants who met thresholds for depression and AUD. Finally, we analyzed association of depression and AUD with socio-demographic variables, and presented risk ratio (RR), 95% confidence interval (CI) and *P* value by using a Generalized Liner Model (GLM). We conducted the analysis using Stata version 13.0 [55]. If a participant was screened positive for both depression and AUD, then that participant was counted as positive for both disorders.

## Results

A total of 1474 adults were screened for depression and AUD. Table 1 provides descriptive statistics of socio-demographic characteristics of the study participants. About two-third (65.8%) of the participants were females; the age of the respondents ranged from 18 years to 83 years with a mean of 39.4 years. One in every six participants (15.9%) was illiterate; a large majority was married (84.5%) and Hindus (84.7%). About a half of the sample was from Brahman/Chhetri caste, followed by Dalit (23.7%) and Janajati (22.2%).

**Table 1:** Percentages met threshold for depression and AUD

	Socio-demographic characteristics of the sample		Percentages met threshold	
	N	%	Depression % (N)	Alcohol Use Disorder % (N)
Sex				
Male	504	34.2	11.3 (57)	19.8 (100)
Female	970	65.8	19.6 (190)	1.1 (11)
Age				
Up to 24	250	17.0	10.8 (27)	4.4 (11)
25–59	1034	70.1	17.2 (178)	7.6 (79)
60 and above	190	12.9	22.1 (42)	11.1 (21)
Education				
Not schooling	235	15.9	36.6 (86)	9.4 (22)
Literate/less than primary	295	20.0	21.1 (62)	6.4 (19)
Primary	312	21.2	11.2 (35)	9.9 (31)
Secondary	546	37.1	10.6 (58)	6.8 (37)
College /University	86	5.8	7.0 (6)	2.3 (2)
Marital status				
Single	126	8.5	10.3 (13)	7.1 (9)
Married	1245	84.5	15.9 (198)	7.3 (91)
Others (widow/divorced/separated)	103	7.0	34.9 (36)	10.8 (11)
Caste/Ethnicity				
Brahman/Chhetri	723	49.0	14.4 (104)	3.6 (26)
Janajati	327	22.2	14.1 (46)	11.6 (38)
Dalit	349	23.7	24.6 (86)	12.6 (43)
Others	75	5.1	14.7 (11)	5.3 (4)
Religion				
Hindu	1249	84.7	17.6 (218)	6.5 (81)
Buddhist	179	12.2	12.3 (22)	16.2 (29)
Others	46	3.1	15.2 (7)	2.2 (1)
Occupation				
Agriculture	906	61.5	17.0 (154)	6.3 (57)
Service/Business	150	10.2	11.3 (17)	10.0 (15)
Unemployed	307	20.8	13.7 (42)	3.9 (12)
Others	111	7.5	30.6 (34.0)	24.3 (27)
Number of family members				
1–4	575	39.0	18.1 (104)	6.6 (38)
5–7	685	46.5	15.9 (109)	7.2 (49)
More than 7 people	214	14.5	15.9 (34)	11.2 (24)
Family income sufficient to manage foods for the period of				
Up to six months	122	8.3	36.1 (44)	9.8 (12)
6–9 months	248	16.8	22.6 (56)	8.1 (20)
9–12 months or more	1104	74.9	13.3 (147)	7.2 (7.9)
Total	1474		16.8 (247)	7.5 (111)

Of the total, 39.9% (males 70.8% and females 23.8%) reported that they consumed alcohol sometimes in their life whereas this percentage was 29.6% (males 55.8% and females 16%) for the past 12 months. Of the total, 16.8% met the threshold for depression and 7.3% for AUD. Percentages of positive screens for depression were higher for females (19.6%) than males (11.3%). On the other hand, the percentage of AUD was higher among males (19.8%) than females (1.1%) (Table 1).

Table 2 presents the risk ratios for depression and AUD from a Generalized Liner Model (GLM). The rates of positive screens for depression and AUD varied based on sex, education, caste/ethnicity, occupation, religion and family income. Results show that females, having no or low level of education, “other” occupations and people with insufficient family income for foods had significantly higher risk of developing depression. By contrast, males, Dalit caste, Buddhists, and participants reporting “other” occupations had significantly higher risk for having AUD.

**Table 2:** Variables associated with depression and AUD

		Likelihood of Depression		Likelihood of AUD	
Variables	N	Adjusted RR (95% CI)	P value	Adjusted RR (95% CI)	*P* value
Sex					
Male (Ref)	504	1	–	1	–
Female	970	1.48 (1.11–1.99)	0.009	0.49 (0.26–0.91)	0.000
Age					
Up to 24 (Ref)	250	1	–	1	–
25–59	1034	1.13 (0.71–1.78)	0.605	1.84 (0.72–0.72)	0.205
60 and above	190	1.05 (0.61–1.82)	0.858	1.19 (0.42–3.38)	0.747
Education					
Not schooling (Ref)	235	1	–	1	–
Literate/less than primary	295	0.62 (0.46–0.82)	0.001	0.70 (0.41–1.19)	0.186
Primary	312	0.36 (0.25–0.53)	0.000	0.63 (0.38–1.04)	0.069
Secondary	546	0.39 (0.27–0.55)	0.000	0.48 (0.28–0.82)	0.007
College /University	86	0.28 (0.12–0.66)	0.004	0.27 (0.07–1.11)	0.069
Marital status					
Single (Ref)	126	1	−/−	1	–
Married	1245	0.87 (0.46–1.64)	0.666	1.14 (0.41–3.18)	0.807
Others (widow/divorced/separated)	103	1.22 (0.58–2.53)	0.600	1.39 (0.43–4.51)	0.586
Caste/Ethnicity					
Brahman/Chhetri (Ref)	723	1	–	1	–
Janajati	327	0.98 (0.67–1.41)	0.896	1.77 (0.93–3.35)	0.080
Dalit	349	1.22 (0.93–0.61)	0.147	2.99 (1.82–4.91)	0.000
Others	75	0.85 (0.49–1.49)	0.571	0.04 (0.37–2.91)	0.937
Religion					
Hindu (Ref)	1249	1	–	1	–
Buddhist	179	0.78 (0.48–1.26)	0.307	2.08 (1.20–3.61)	0.009
Others	46	0.68 (0.37–1.27)	0.231	0.24 (0.04–1.44)	0.119
Occupation					
Agriculture (Ref)	906	1	–	1	–
Service/Business	150	1.00 (0.63–1.58)	0.994	1.22 (0.72–2.06)	0.454
Unemployed	111	1.01(0.73–1.41)	0.930	1.50 (0.79–2.87)	0.218
Others	307	1.72 (1.22–2.41)	0.002	2.11 (1.37–3.24)	0.001
Number of family members					
1–4 (Ref)	575	1	–	1	–
5–7	685	0.88 (0.69–1.12)	0.311	1.07 (0.73–1.56)	0.727
More than 7 people	214	0.92 (0.57–1.30)	0.637	1.29 (0.85–1.96)	0.238
Family income sufficient to manage foods for the period of					
Up to Six months (Ref)	122	1	–	1	–
6–9 months	248	0.88 (0.62–1.25)	0.474	1.25 (0.69–2.28)	0.467
9–12 months or more	1104	0.60 (0.44–0.83)	0.001	1.23 (0.55–2.14)	0.458

## Discussion

The results indicated that one in every 6 people (16.8%) attending primary health care services in Chitwan district had depression and one in every 14 (7.5%) had AUD. When evaluating by gender, one out of five women (19.6%) had depression and one out of five (19.8%) men had AUD. As can be among a population of health care attendees, these rates are higher than the prevalence rates reported in the community survey (depression =11.2% and AUD =5%) in Chitwan [38]. However, these rates are lower than the rates found among the population affected by conflict in Chitwan, Tanahu and Dang [32], Jumla [31], among Bhutanese refugees [35], earthquake affected populations [56] and persons seeking specialty care for non-communicable diseases such as hypertension [40] and diabetes [39].

We found significant association between a number of socio-demographic variables with both depression or AUD. Females have a 1.5 greater risk of depression compared to males, which is consistent with previous studies in Nepal [32, 33, 57–59] and other LMICs [60–62]. On the other hand, females were less likely to have AUD than males, again consistent with the study conducted among the general population in Chitwan [58], Bhutanese refugees [35], people living in the squatter settlements of Kathmandu Valley [63], the population affected by the earthquake [56], and other LMICs [25, 64, 65]. Traditionally, females in Nepal drink less than males; therefore, males have higher risk for alcohol use disorder [35, 66]. Similarly, Dalit castes have greater risk for AUD than Brahman/Chhetri, which could be due to the fact that, historically, Brahman/Chhetri castes do not drink alcohol, as opposed to Janajati and Dalit caste, for which drinking alcohol is culturally acceptable [67]. Moreover, people having any level of education have lower risk for depression compared to those with no education; however, only those with secondary level of education have lower risk for AUD compared to those with no education. In addition to this, the reason for a lack of association between college/university level of education and AUD could be the fact that only a few participants with AUD (*N* = 2) have college/university level of education. Finally, participants reporting “other” occupations and those with insufficient family income to manage foods for more than six months in a year have greater risk for depression. Our finding regarding greater risk of depression among occupational groups is also consistent with our prior studies using community samples [32, 59].

This study found no association between depression and age of the participants which contrasts with previous studies [31, 32], where older age was significantly associated with depression. This is likely due to the fact that community based studies likely include more healthy young/middle-age adults, whereas young/middle-age adults in primary care are biased toward those with physical health problems. Multivariate analysis also suggests no association between marital status and depression which also contrasts with the findings of previous studies [31, 32], where widows/widower/separated had greater risk for depression than their married and single counterparts. This may also be due to confounding factors related to who is more likely to seek primary care services, i.e., older adults with greater burdens of physical health problems.

The findings of this study may have several implications; especially for making sure that mental health services are accessible to those who have greater risk for depression or AUD. First, the study has provided an indication on the prevalence of depression and AUD among people attending primary health care clinics. As a significant proportion of the patients attending primary care reported depression or AUD; this could be useful for policy makers and mental health professionals to estimate the amount of human resources and time needed to dedicate depression/AUD care in the primary health care clinics. Moreover, the results of this study could be also useful to estimate drugs needed for depression and AUD treatment. Second, one out of five women attending primary care services have reported depression and one out of five men have AUD; the existing human resources may be inadequate to address this; therefore, placing midlevel mental health workers at the health facility could be transformative in addressing this burden of mental health problems. The district mental health care plan (MHCP) which was developed, implemented and evaluated in Chitwan [47] has put in place the community counselors as mid-level health workers to provide focus psychosocial counseling, especially the Healthy Activity Program (HAP) and Counseling for Alcohol Problems (CAP). Moreover, a trial evaluating the added value of community counselors for providing mental health treatments is underway. Third, previous study indicated that only 1.3% people with AUD and 1.8% with depression receive treatment from primary health care facilities in Chitwan [38]; the PHQ-9 and AUDIT could be feasibly administered in the routine health system to improve detection of depression and AUD. However, considering the limited number of existing health care workers, universal screening may not be feasible in Nepal. The PHQ-9 validation in primary care settings in Nepal included a 2-part tiered algorithm that first screens for a local idiom “heart-mind problems” associated with functional impairment, then is followed by the full PHQ-9 for those who screen positive. We have found that with an algorithm comprising of two screening questions (i.e. presence of heart-mind problems and function impairment due to heart-mind problems) to determine who should receive the full PHQ-9, the number of patients requiring administration of the PHQ-9 could be reduced by 50%, PHQ-9 false positives would be reduced by 18%, and 88% of patients with depression would be correctly identified [52]. Therefore, the heart-mind problems screener could be an effective strategy to detect people with depression in the routine health care system along with PHQ9.

Fourth, the results indicated a significant association between depression and AUD and socio-demographic characteristics of the participants. Future intervention, especially awareness and sensitization program, should be targeted to those sub-population who are more at risk for having depression and AUD. Finally, due to labor/education migration, a large number of males are outside of the country and the females’ population is pre-dominantly high in most part of the country. In Nepal, generally females are engaged in the household level activities such as cooking, taking care of children and looking after cattle; therefore, it is often challenging for females to travel to another district for treatment of their mental health conditions. Further, the prevalence of depression is also very high among females; therefore integration of mental health services into the primary health care system could be an appropriate strategy to make sure that mental health services are accessible to those having problems with travel for seeking health care.

Several limitations should be considered in interpreting the results. First, the study sites were chosen for implementation and evaluation of PRIME mental health care plan, and were not intended to represent a specific cultural context or geographical territory in Nepal. Second, we relied on self-reports which has been shown to predict inflated rates of mental health problems [68]. The PHQ-9 has a false positive rate of approximately 4–6 false positive depression cases per 10 primary care patients identified with PHQ-9 scores above cut-offs [69, 70]. In Nepal, the false positive rate with the PHQ-9 is also approximately 6 false positives per 10 patients screening positive for depression [52], with fewer than one per hundred false negatives. This also points towards the need to minimize the risk of false positives in clinical programs, such as through the use of tiered algorithms and appropriate clinical supervision. Third, cultural factors might have influenced AUDIT scores. For example, women or high caste people are not socially sanctioned to drink; they might have underreported their drinking. Fourth we included adults (age 18 years or above) only in the study so it does not provide any information about children/adolescents’ mental health; nevertheless, they are the key population receiving services from primary health care clinics in Nepal. Fifth, there was significantly high proportion of female participant in the sample. The large proportion of the female sample in the study could be explained by a high out-migration (mainly to Gulf countries) of male populations in the area of study. If we look at the out-migration trend in Nepal over the census period, of the total population, the 1991 census recorded an absent population of 3.4% (658,290,of which 83.2% were male and 16.8% were female); the 2001 census recorded an absent population of 3.2% (762,181, of which 89.2% were male and 10.8% were female) and the 2011 census recorded an absent population of 7.3% (1,921,494, of which 87.6% were male and 12.4% were female) [71]. Finally, the specialists’ mental health services are available in both district hospital and private hospitals; therefore, the prevalence of depression and alcohol use disorder could be even higher in other districts where the availability of mental health services is more limited than in Chitwan.

## Conclusion

This study shows that depression (16.8%) and alcohol use disorders (7.3%) are common among adults attending primary health care services in Nepal. The rates found in this study are higher than the prevalence rates reported in the community sample in the same population. Very few people (1.3% people with AUD and 1.8% with depression) receive treatment from primary health care facilities in Chitwan [38]. Detection and treatment of people with mental health problems in primary health care clinics has been recommended as one of the effective strategies to minimize treatment gap; therefore, we argue for the importance of mental health training to primary health care workers in detection and initiation of evidence based treatment in primary health care settings. In Nepal, the existing human resources in primary care may be inadequate to address high burden of mental health problems; therefore, placing midlevel mental health workers at the health facility could be transformative in addressing this burden.

## Abbreviations

AUD: Alcohol Use Disorder
AUDIT: Alcohol Use Disorder Identification Test
HP: Health Post
LMICs: Low and Middle Income Countries
PHC: Primary Health Care
PHCC: Primary Health Care Center
PHQ9: Patients Health Questionnaire-9
PRIME: PRogramme for Improving Mental health carE
SHP: Sub-Health Post
TPO: Transcultural Psychosocial Organization
